# Bioactive Fractions from *Bougainvillea* × *buttiana* Holtum & Standl (var. Rose): Antioxidant, Anti-Inflammatory, Enzyme Inhibitory and Cytoprotective Effects Against Oxidative Stress

**DOI:** 10.3390/molecules31132389

**Published:** 2026-07-07

**Authors:** Vera L. Petricevich, Luis Martínez-Cuevas, Mayra Cedillo-Cortezano, Gabriela Castañeda-Corral

**Affiliations:** Facultad de Medicina, Universidad Autónoma del Estado de Morelos (UAEM), Calle Leñeros, Esquina Iztaccíhuatl s/n Col. Volcanes, Cuernavaca C.P. 62350, Morelos, Mexico; vera.petricevich@uaem.mx (V.L.P.); luis_123b@hotmail.com (L.M.-C.); mayra.cedillo@docentes.uaem.edu.mx (M.C.-C.)

**Keywords:** phenolic compounds, antioxidant activity, anti-inflammatory activity, antidiabetic potential, cytoprotective activity

## Abstract

Background: *Bougainvillea* species have been used in traditional Mexican medicine, but their bioactive compounds and mechanisms of action are insufficiently studied. This is the first comprehensive evaluation of fractions from the acetone extract of *Bougainvillea* × *buttiana* Holtum & Standl (var. Rose), combining phytochemical profiling with in vitro multitarget bioactivity assessment. Methods: Eleven fractions were analyzed for total phenolic (TPC) and flavonoid content (TFC) and antioxidant capacity using the DPPH assay. The most active fractions were further tested for nitric oxide (NO) scavenging, protection of erythrocytes and bovine serum albumin (BSA) from oxidative damage, inhibition of enzymes involved in inflammation (PLA_2_, COX, LOX) and carbohydrate metabolism (α-glucosidase, α-amylase, tyrosinase), cytoprotective effects in L929 fibroblasts exposed to hydrogen peroxide, and their main metabolites were qualitatively identified by HPLC-UV-Vis. Results: All fractions showed significant TPC and TFC and concentration-dependent antioxidant activity. The fractions with the highest antioxidant indices were F5, F7, and F9. These effectively scavenged NO, protected erythrocytes and L929 cells (maintaining viability at 82.0%, 75.6%, and 72.0%, respectively), and inhibited key enzymes. Seven major compounds, mainly flavonoids, were identified. Conclusions: These findings showed that flavonoid-enriched fractions from *B.* × *buttiana* exhibit coordinated antioxidant, anti-inflammatory, antidiabetic, and cytoprotective effects, suggesting potential to treat oxidative stress-related disorders.

## 1. Introduction

Chronic diseases are long-term conditions that require continuous management and are a leading cause of disability and death worldwide [1,2]. While their exact causes are not fully understood, oxidative stress and chronic inflammation are key factors in disease development [3]. Cardiovascular disease, cancer, and diabetes account for most global deaths and contribute significantly to healthcare costs. In low- and middle-income countries, limited access to effective treatments often leads to greater reliance on medicinal plants as alternative therapies due to the high cost and limited availability of conventional medicines (50%) [4,5].

Throughout history, plants have remained a valuable source of new drugs. Their therapeutic effects are mainly due to structurally diverse secondary metabolites, which are present at lower concentrations than macromolecules [6,7]. Polyphenols, secondary metabolites present in plant-based foods, provide important health benefits, and their regular consumption has been associated with a reduced risk of chronic diseases [8,9]. These compounds are classified as either flavonoids or non-flavonoids, with flavonoids representing the predominant group [10]. In recent years, plant-derived phenolic acids and flavonoids have attracted considerable interest due to their antioxidant, anti-inflammatory, antidiabetic, and cardioprotective properties [9,11]. Given their therapeutic potential, it is essential to identify bioactive compounds and elucidate their mechanisms of action in physiological systems. In this context, bioguided fractionation plays a critical role in isolating compounds with pharmacological potential [12]. Extracts and fractions are systematically screened for biological activity before chemical characterization. High-performance liquid chromatography (HPLC) enables the detection of bioactive metabolites and supports the identification of both known and novel compounds, thereby contributing to drug discovery [13]. Numerous studies have employed HPLC to analyze medicinal and food species, revealing chromatographic profiles abundant in glycosylated and non-glycosylated secondary metabolites [14]. Beyond identifying individual compounds, quantification of total phenolic content (TPC) and total flavonoid content (TFC) helps to estimate the extract’s chemical richness, and these values often correlate with in vitro antioxidant activity, typically evaluated by neutralizing reactive oxygen species (ROS) and reactive nitrogen species (RNS) [15,16]. Moreover, several studies reported that phenolic compounds can also inhibit the activity of key enzymes involved in inflammation, carbohydrate metabolism, and skin pigmentation. Flavonoids such as quercetin, kaempferol, and caffeic acid have been described as natural modulators of these processes [9,17].

*Bougainvillea* is a genus of ornamental plants characterized by colorful bracts, which are frequently mistaken for petals. Originally native to South America, *Bougainvillea* was later introduced to Mexico [18]. The most common species include *Bougainvillea spectabilis* Willd, *Bougainvillea glabra* Choisy, and *Bougainvillea* × *buttiana* Holttum and Standl. (var. Rose) [18,19,20]. In traditional Mexican medicine, the *Bougainvillea* genus has been used to treat respiratory and gastrointestinal disorders, coughs, asthma, bronchitis, and nausea, as well as for wound healing [18,19]. In support of these traditional uses, several studies have demonstrated that extracts from these plants exhibit diverse biological activities with therapeutic potential [18,19,20]. For example, *B. spectabilis* root bark extract has exhibited hypoglycemic effects in diabetic rats [21,22]. Similarly, our research group has shown that *Bougainvillea* × *buttiana* (var. rosa) bract extracts display antioxidant, anti-inflammatory, antinociceptive, and wound-healing properties [23,24,25,26]. These effects are primarily attributed to its rich profile of secondary metabolites, such as betalains, flavonoids, and phenolic compounds [23,24,25,26,27]. Accordingly, polyphenols and flavonoids with demonstrated anti-inflammatory activity have been previously identified in this species [26,28]. Specifically, we demonstrated that the extracts of *B.* × *buttiana* exhibit anti-inflammatory properties in vitro and in vivo, following both systemic and topical administration, potentially mediated by COX inhibition [25]. However, the underlying mechanisms remain poorly understood. Despite growing evidence supporting the antioxidant and anti-inflammatory properties of *Bougainvillea* species, most studies have focused on crude extracts, limiting the identification of specific bioactive fractions and their molecular targets. Therefore, the present study aimed to (i) perform bio-guided fractionation of the acetone extract of *Bougainvillea* × *buttiana*, (ii) determine the total phenolic content (TPC) and the total flavonoid content (TFC), (iii) identify fractions with relevant antioxidant and anti-inflammatory activity using complementary in vitro models, and (iv) explore their preliminary effects on enzymes involved in inflammation and carbohydrate metabolism, and evaluate their cytoprotective effects against oxidative stress, supported by chromatographic qualitative analyses. In addition, given the in vivo anti-inflammatory studies and wound-healing properties reported in our previous work, this study aims to identify bioactive fractions with potential applications in complementary and orthomolecular medicine.

## 2. Results

The acetone extract of *B.* × *buttiana* was fractionated using a bioassay-guided approach based on the procedure previously described by Martínez-Cuevas et al. (2025) [24]. The fractionation was guided by in vivo anti-inflammatory and wound-healing activities, yielding 11 fractions designated F-1 through F-11.

### 2.1. Quantification of the Total Phenolic Content and the Total Flavonoid Content

**Figure 1:**
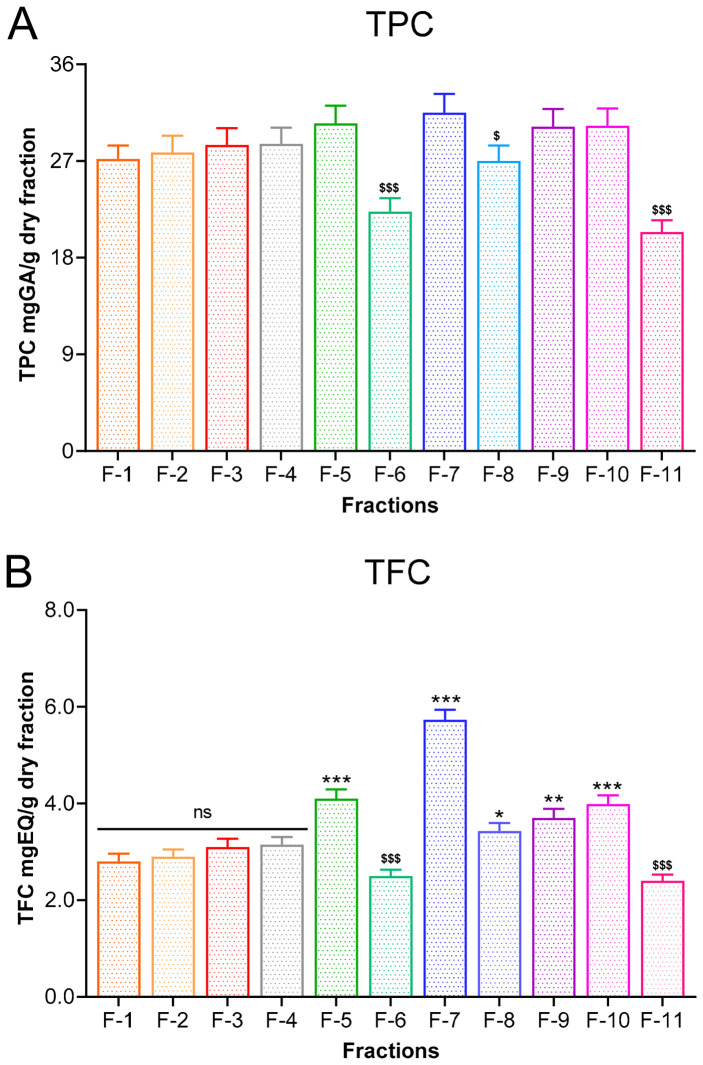
Fractions F1–F11, derived from the acetone extract of *B.* × *buttiana*, contain high levels of phenolic compounds and flavonoids. (**A**) Total phenolic content (TPC) is expressed as milligrams of gallic acid equivalents (mg GAE) per gram of dry fraction. (**B**) Total flavonoid content (TFC) is expressed as milligrams of quercetin equivalent (mg QE) per gram of dry fraction. Data are shown as mean ± SD from three independent experiments. * *p* < 0.05, ** *p* < 0.01, *** *p* < 0.001 vs. F-1; $ *p* < 0.05, $$$ *p* < 0.001 vs. F-7, by one-way ANOVA followed by the Tukey test. ns: not significant.

Phenolic compounds and flavonoids are secondary metabolites well established for their antioxidant properties [29]. Based on previous findings regarding the antioxidant activity of *B.* × *buttiana* extracts, the Total Phenolic Content (TPC) and Total Flavonoid Content (TFC) were assessed in fractions F-1 through F-11. Quantitative analyses revealed that all fractions contained substantial concentrations of these compounds. As expected, TPC values exceeded TFC values, since flavonoids represent a subclass of phenolic compounds. Notably, most fractions exhibited similar levels of TPC, except for F-6, F-7, and F-8, which showed reduced phenolic content (Figure 1A). In contrast, F-5, F-7, and F-10 displayed significantly higher TFC, while F-1 to F-5, F-8, and F-9 exhibited comparable flavonoid content. F-6 and F-11 had the lowest total flavonoid levels (Figure 1B). These results highlight variability among fractions and suggest that specific fractions exhibit particularly high biological activity, as previously reported for *B.* × *buttiana* extracts.

### 2.2. Antioxidant Activity of Fractions F-1 to F-11 in the 2,2′-Diphenyl-1-Picrylhydrazine (DPPH) Radical Scavenging Assay

**Figure 2:**
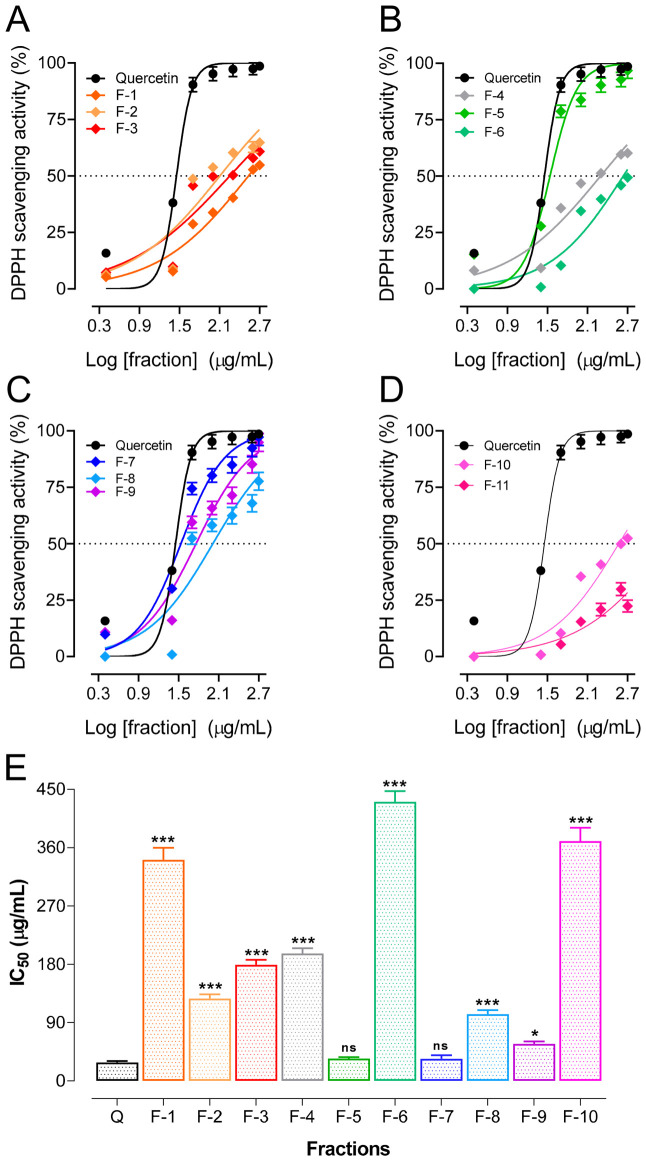
Fractions F1–F11, derived from the acetone extract of *B.* × *buttiana,* exhibit concentration-dependent antioxidant capacity. (**A**–**D**) display concentration-response curves for DPPH radical scavenging activity. (**E**) presents IC_50_ values derived from these curves. Each data point or bar represents the mean ± SD from three independent experiments. Dashed lines in (**A**–**D**) show 50% of the DPPH scavenging activity, which is used to calculate the IC_50_. * *p* < 0.05, *** *p* < 0.001 vs. Quercetin (Q) by one-way ANOVA followed by the Dunnett test. ns: not significant.

In this study, the DPPH assay was used to evaluate the antioxidant capacity of fractions F-1 to F-11. Reduction of purple DPPH to yellow diphenylpicrylhydrazine indicated free radical scavenging activity [30]. Fractions F-1 to F-11 showed concentration-dependent antioxidant activity (Figure 2A–D). At 500 µg/mL, the antioxidant activity of the fractions ranged from 52.4% (F-6) to 97.2% (F-7), except for F-11, which showed only 22.4% activity and was therefore excluded from further analysis. Fractions F-5, F-7, and F-9 achieved maximum DPPH scavenging activities of 96.8%, 97.2%, and 94.9%, respectively, which were comparable to the reference standard quercetin (98.3%). IC_50_ values, calculated from the concentration-response curves, ranged from 34.26 µg/mL (F-7) to 370.2 µg/mL (F-6). It is important to note that the IC_50_ values of fractions F-5 and F-7 were not statistically significantly different from that of quercetin, indicating that they have the same potency. Similarly, the IC_50_ value of fraction F-9 was similar to that of quercetin. In contrast, the IC_50_ values of the other fractions were significantly higher than those of the control (Figure 2E). These results demonstrate that fractions F-5, F-7, and F-9 exhibit the highest antioxidant activity in the DPPH assay.

#### 2.2.1. Antioxidant Activity Index (AAI) and Relative Antioxidant Yield (RAY)

**Figure 3:**
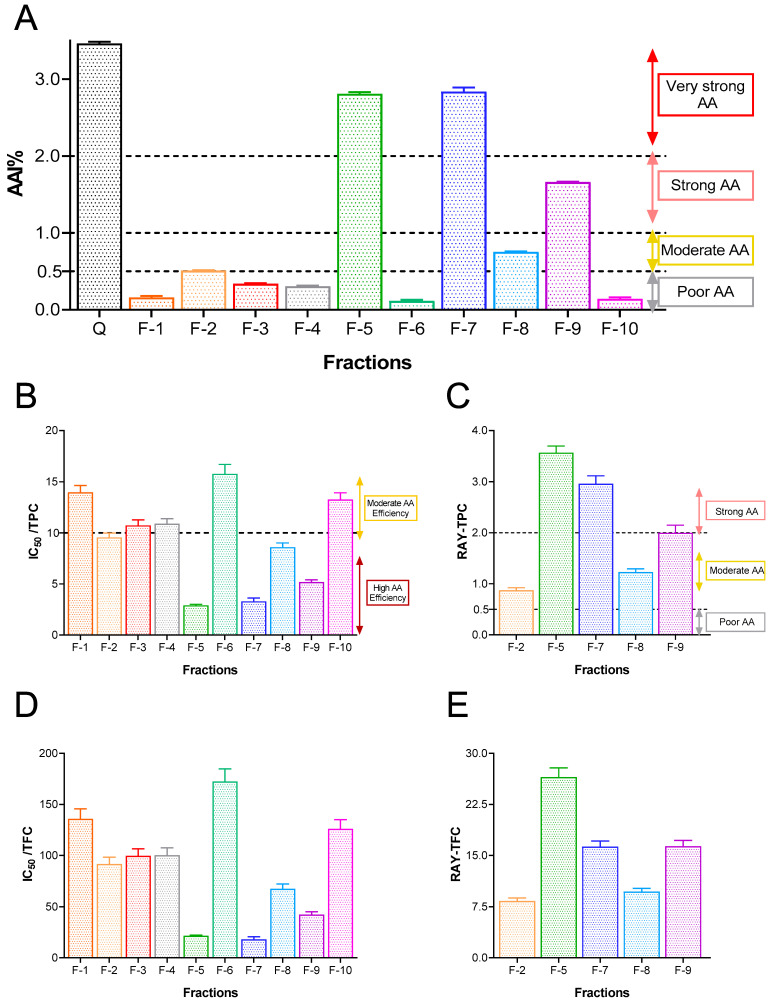
Analysis of the efficiency of antioxidant activity. (**A**) Antioxidant activity index (AAI). (**B**,**D**) relationship between the IC_50_ values and the TPC (IC_50_/TPC) and the TFC (IC_50_/TFC), respectively. (**C**,**E**) Relative Antioxidant Yield (RAY) relationship between the AAI × 100 values and TPC, and TFC. Each bar represents the mean ± SD from three independent measurements.

Furthermore, to determine the antioxidant capacity of the fractions and select those with higher bioactivity potential, the Antioxidant Activity Index (AAI), the IC_50_/TPC and IC_50_/TFC ratios, and the Relative Antioxidant Yield (RAY) ratio relative to TPC or TFC were calculated. The AAI is a standardized parameter derived from the DPPH assay, defined as the ratio of the final DPPH solution concentration to the IC_50_ value for each sample. This index enables a comparative assessment of antioxidant strength. According to Scherer and Godoy (2009) [31], AAI values < 0.5 indicate low antioxidant activity; values between 0.5 and <1.0 indicate moderate activity; values between 1.0 and 2.0 indicate strong activity; and values > 2.0 indicate very strong activity. Based on these criteria, F-1, F-3, F-4, F-6, and F-10 showed poor activity, F-2 and F-8 showed moderate activity, F-9 showed strong activity, and F-5 and F-7 showed very strong activity (Figure 3A). Moreover, IC_50_ ratios normalized to TPC (IC_50_/TPC) and TFC (IC_50_/TFC) were calculated to assess antioxidant efficiency. These ratios represent the amount of phenolic or flavonoid compounds needed to achieve 50% antioxidant activity. Lower IC_50_/TPC or IC_50_/TFC values indicate greater antioxidant efficiency per unit of compound, compared with absolute IC_50_ values. IC_50_/TPC ratios below 10 reflect high efficiency, values from 10 to 30 indicate moderate efficiency, and values above 30 indicate low efficiency. The analysis showed IC_50_/TPC ratios ranged from 2.91 (F-5) to 15.79 (F-6) and IC_50_/TFC ratios ranged from 21.7 (F-5) to 172.5 (F-6), indicating substantial variability in antioxidant efficiency among the fractions (Figure 3B,D). Fractions F-2, F-5, F-7, F-8, and F-9 confirmed high antioxidant efficiency, while F-1, F-3, F-4, F-6, and F-10 showed moderate efficiency. Combined analysis of the Antioxidant Activity Index (AAI) and IC_50_/TPC ratios identified F-2, F-5, F-7, F-8, and F-9 as having the greatest antioxidant potential. To validate these findings, the ratio of Relative Antioxidant Yield (RAY) was calculated and normalized to TPC (RAY-TPC) and TFC (RAY-TFC) (Figure 3C,E). Lower RAY values correspond to higher antioxidant performance. A RAY-TPC ratio greater than 2.0 indicates high efficiency, 0.5–2.0 indicates moderate efficiency, and values below 0.5 represent low efficiency. Fractions F-2 and F-8 were classified as moderate, whereas F-5, F-7, and F-9 demonstrated high efficiency, indicating superior antioxidant performance per unit of phenolic and flavonoid compounds. The highest RAY-TPC and RAY-TFC values were obtained with F-5. Based on the combined analysis of antioxidant indices (AAI, IC_50_/TPC ratios, and RAY values), qualitative phytochemical composition (this study), as well as the in vivo anti-inflammatory and wound-healing activities reported in our previous study [24], fractions F-5, F-7, and F-9 were selected for further biological evaluation and HPLC-UV-Vis analyses. Since nitric oxide is closely associated with oxidative stress and inflammatory pathways, these fractions were additionally assessed using the nitric oxide (NO) radical-scavenging assay.

#### 2.2.2. Effect of F-5, F-7 and F-9 on Nitric Oxide Scavenging

**Figure 4:**
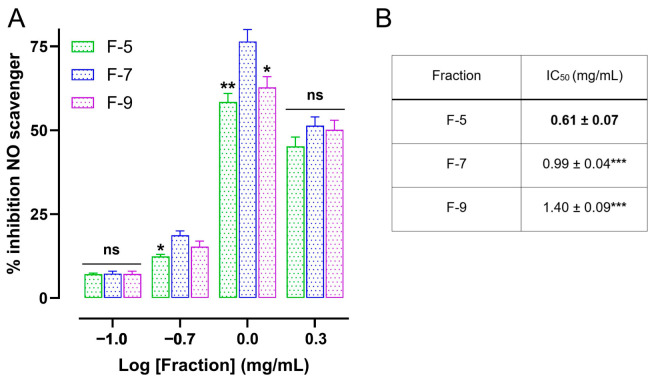
Fractions F-5, F-7, and F-9 reduced nitric oxide scavenging in a concentration-dependent manner. (**A**) Percentage inhibition of nitrite formation calculated relative to the control at 180 min. (**B**) IC_50_ values. Data are shown as mean ± SD (*n* = 3). Bars: * *p* < 0.05, ** *p* < 0.01, by two-way ANOVA followed by the Tukey test. Just a statistical comparison vs. F-7 is shown. ns: no significant. Table. *** *p* < 0.001 vs. F-5, by one-way ANOVA followed by the Dunnett test.

Based on the combined analysis of antioxidant indices (AAI, IC_50_/TPC ratios, and RAY values), qualitative phytochemical composition, and previously reported in vivo anti-inflammatory and wound-healing activities, fractions F-5, F-7, and F-9 were selected for further biological evaluation. Since nitric oxide is closely associated with oxidative stress and inflammatory pathways, these fractions were additionally assessed using the nitric oxide (NO) radical-scavenging assay. Under anoxic conditions, sodium nitroprusside spontaneously generates NO, which reacts with molecular oxygen (O_2_) to form nitrite ions, which are detected with the Griess reagent [32,33]. In this assay, lower NO production indicates greater scavenging activity of the test sample. Fractions F-5, F-7, and F-9 exhibited a concentration-dependent increase in NO-scavenging activity compared to the control. The highest inhibition percentages reached 76.5%, 62.8%, and 58.4% for F-5, F-7, and F-9, respectively (Figure 4A). Additionally, the concentration appropriate to produce 50% NO radical scavenging (IC_50_) indicated that F-5 possessed the most potent antioxidant activity. Overall, the potency ranking was F-5 > F-7 > F-9 (Figure 4B).

### 2.3. In Vitro Anti-Inflammatory Activity of Fractions F-5, F-7, and F-9: Effects on Erythrocyte Membrane Stabilization, BSA Denaturation, and Trypsin Activity

**Figure 5:**
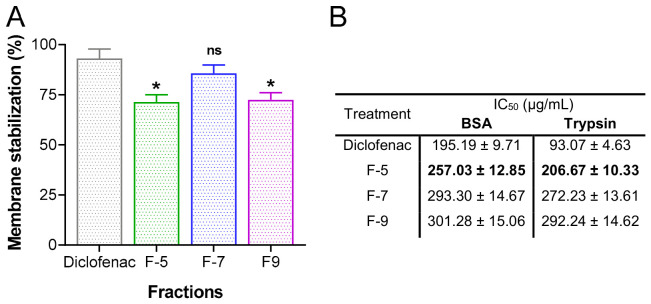
F-5, F-7, and F-9 displayed in vitro anti-inflammatory properties. (**A**) Erythrocyte membrane stabilization assay. (**B**) IC_50_ values of the fractions in BSA-inhibited denaturation and trypsin activity assays. Data are shown as mean ± SD. * (*p* < 0.05) compared to diclofenac, by one-way ANOVA followed by the Dunnett test. ns: not significant.

The anti-inflammatory activity of fractions F-5, F-7, and F-9 was assessed using three in vitro assays: erythrocyte membrane stabilization, inhibition of heat-induced bovine serum albumin (BSA) denaturation, and inhibition of trypsin activity. At a concentration of 400 µg/mL, F-5, F-7, and F-9 inhibited erythrocyte hemolysis by 71.40%, 85.70%, and 72.48%, respectively. The inhibitory effect of F-7 was statistically comparable to the positive control, diclofenac (96.8% at 100 µg/mL), whereas F-5 and F-9 exhibited significantly lower activity (*p* < 0.05). These results indicate that F-7 produced strong membrane-stabilizing activity (Figure 5A). All fractions also significantly inhibited BSA denaturation and trypsin activity (Figure 5B). F-5 exhibited the lowest IC_50_ values in both assays, indicating the highest potency, whereas F-7 and F-9 showed similar IC_50_ values. Nevertheless, all fractions displayed slightly lower activity than diclofenac (Figure 5B). These results highlight F-5 as the most potent fraction in the anti-inflammatory in vitro assays.

### 2.4. Effect of Fractions F-5, F-7, and F-9 on the Activity of Inflammation- and Diabetes-Related Enzymes

Oxidative stress and inflammation are closely associated with the onset and progression of several chronic diseases, including diabetes mellitus. In this context, the biological activities of the selected fractions were evaluated by assessing their effects on key enzymes involved in both inflammatory processes and glucose metabolism. Regarding inflammation, the fractions were tested against phospholipase A_2_ (PLA_2_), cyclooxygenases (COX-1 and COX-2), and lipoxygenase (LOX), enzymes that play central roles in the synthesis of eicosanoids, including prostaglandins and leukotrienes, well-recognized mediators of inflammatory pathways [34]. Regarding glucose metabolism, the fractions were evaluated for their effects on α-amylase and α-glucosidase, key enzymes in carbohydrate digestion [35], whose inhibition is a well-established therapeutic strategy for controlling postprandial hyperglycemia in type 2 diabetes mellitus [36]. Furthermore, since tyrosinase is involved in skin pigmentation, the effects of the fractions on this enzyme were also examined (Table 1).

Enzyme inhibition assays revealed that all three fractions inhibited the activity of the tested enzymes, each demonstrating a distinct activity profile (Table 1). For PLA_2_ (hG-IIA and pG-IB), F-5 was the most potent, exhibiting the lowest IC_50_ values; indomethacin was used as the positive control (IC_50_ 91 ± 4.7 µg/mL). Regarding COX isoforms, F-7 demonstrated the highest inhibitory potency, followed by F-9 and F-5. However, all fractions were less potent than the reference drug diclofenac (IC_50_: 140 ± 7.4 µg/mL for COX-1; 78 ± 3.9 µg/mL for COX-2). All fractions preferentially inhibited COX-2 over COX-1, as indicated by COX-1/COX-2 selectivity index (SI) values greater than 1 (F-5: 3.6; F-7: 2.7; F-9: 2.9). For LOX inhibition, IC_50_ values ranged from 98.30 to 120.70 µg/mL, with F-5 and F-7 exhibiting the highest and statistically comparable inhibitory activity, with F-9 showing the lowest inhibitory activity among the three fractions.

Regarding enzymes involved in carbohydrate digestion, all three fractions inhibited α-amylase with IC_50_ values comparable to acarbose (11.42 µg/mL), with F-7 showing the greatest potency, followed by F-9 and F-5. For α-glucosidase, F-7 (IC_50_ = 12.3 µg/mL) and F-9 (IC_50_ = 12.2 µg/mL) exhibited higher inhibitory potency than acarbose (IC_50_ = 14.8 µg/mL), while F-5 was the least potent (IC_50_ = 16.8 µg/mL). For tyrosinase, moderate inhibition was observed across all fractions, with F-7 (IC_50_ = 76.50 µg/mL) being the most active, though all fractions were markedly less potent than the reference drug kojic acid (IC_50_ = 12.50 ± 0.53 µg/mL).

Overall, F-7 exhibited the broadest inhibitory profile, with the highest potency against most of the tested enzymes, reflecting its potential as a multi-target bioactive fraction. F-5 was the most effective against PLA_2_ isoforms and showed marked inhibition of COX-1, COX-2, and LOX, indicating that modulation of these enzymes contributes significantly to its anti-inflammatory activity. F-9 displayed intermediate potency, with relevant inhibition of α-glucosidase and moderate effects on COX and tyrosinase. Taken together, these results highlight the multi-target inhibitory potential of the three fractions and support further investigation into their bioactive constituents.

### 2.5. Cytoprotective Effects Against H_2_O_2_-Induced Oxidative Stress in L929 Fibroblasts

**Figure 6:**
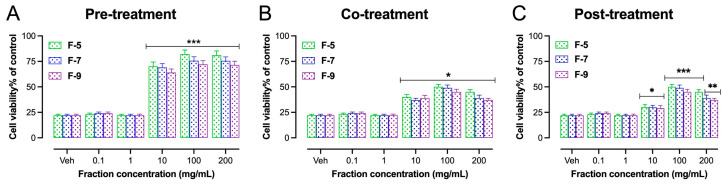
Effect of F-5, F-7, and F-9 fractions on cell viability. The percentage of survival of L929 cells treated with extracts in different conditions, pre-, co-, and post-treatments with H_2_O_2_ for 24 h, was calculated as described in Section 4. Data are expressed as the mean ± SD (*n* = 20).* *p* < 0.05; ** *p* < 0.01; *** *p* < 0.001, by two-way ANOVA followed by the Tukey test. Only the statistical comparison vs. vehicle (Veh) is shown.

To further investigate the biological relevance of the antioxidant activity observed in the chemical assays, the cytoprotective effects of fractions F-5, F-7, and F-9, at a concentration range between 0.1 and 200 μg/mL, were evaluated in L929 murine fibroblasts exposed to hydrogen peroxide (H_2_O_2_)-induced oxidative stress. Hydrogen peroxide is widely used as an in vitro model to generate ROS, leading to oxidative damage, mitochondrial dysfunction, and reduced cell viability. L929 cells were treated with 1 mM H_2_O_2_ as a model of oxidative stress, and this caused a decrease in cell viability by around 75% after 24 h of exposure. As shown in Figure 6, the potential antioxidant effect of the fractions was assessed as pre-, co-, and post-treatment conditions. Pre-treatment with the fractions provided the best cytoprotective effect, preserving cell viability at 82.0%, 75.6%, and 72.0% for the F-5, F-7, and F-9 fractions at 100 and 200 μg/mL, respectively (Figure 6A). In contrast, co- and post-treatment with H_2_O_2_ resulted in only modest protection across all fractions (Figure 6B,C). Among the three treatment protocols, the greatest protective effect of F-5, F-7, and F-9 at 10 and 100 µg/mL was observed when the fractions were administered before H_2_O_2_ exposure. These results indicate that pretreatment with the selected fractions confers superior protection against H_2_O_2_-induced oxidative stress, supporting their potential role as preventive cytoprotective agents. The observed differences may be attributed to variations in the concentration and composition of phenolic and flavonoid constituents among the fractions.

### 2.6. Chromatographic Profile by HPLC-UV-Vis

**Figure 7:**
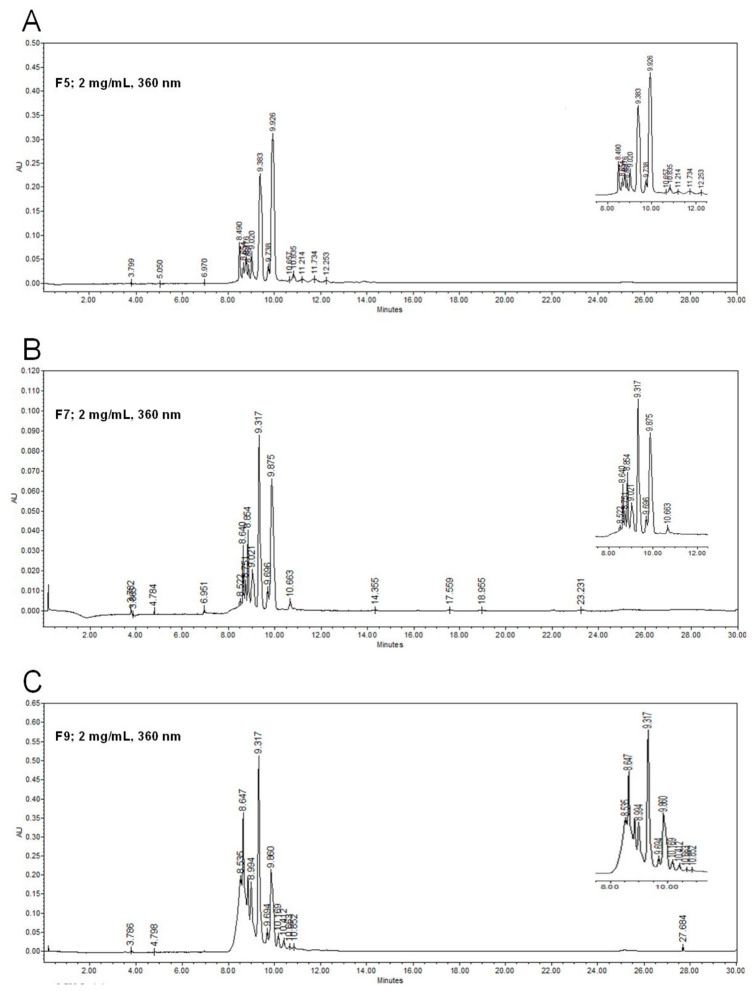
HPLC chromatographic profiles of fractions F-5 (**A**), F-7 (**B**), and F-9 (**C**). Inserted into the right corner of each panel is a magnification of the peaks shown between 8 and 12 min.

Fractions F-5, F-7, and F-9, previously identified as the most bioactive samples based on their elevated total phenolic content (TPC), total flavonoid content (TFC), and in vitro biological activities, were subjected to HPLC-UV-Vis analysis for tentative qualitative phytochemical characterization. The recovery yields obtained after fractionation were 32.16% for F-5, 26.78% for F-7, and 26.90% for F-9. Material loss during the chromatographic process may be associated with adsorption of analytes to the stationary phase, incomplete elution, or partial degradation of labile constituents, phenomena commonly reported during preparative separations [20]. The chromatographic profiles revealed complex metabolite distributions among the three fractions. Fraction F-5 showed 16 detectable peaks with retention times ranging from 3.79 to 12.25 min (Figure 7A). Fraction F-7 presented 17 peaks distributed between 3.78 and 23.23 min (Figure 7B), whereas F-9 exhibited 13 peaks with retention times from 3.78 to 27.68 min (Figure 7C).

#### Qualitative Profiling and Relative Composition of Bioactive Fractions by HPLC-UV-Vis

The broader retention time ranges observed for F-7 and F-9 suggest the presence of compounds with greater polarity diversity compared with F-5. In contrast, the narrower elution window of F-5 may indicate enrichment in metabolites of similar polarity, which could partially explain its consistent antioxidant and cytoprotective performance.

Major constituents were tentatively identified by comparing retention times and UV-Vis absorption spectra with available reference standards and literature data. The detected compounds were predominantly consistent with phenolic acids and flavonoid derivatives, supporting the chemical basis of the biological activities observed in the selected fractions.

Comparative profiling based on relative peak area percentages showed that fractions F-5 and F-7 shared seven major metabolites (Table 2):

2,5-dihydroxybenzoic acid (2,5DHB), myricetin 3-*O*-β-D-glucopyranoside (M3G), chlorogenic acid (CGA), quercetin-3-rutinoside (Q3Rut), quercetin-3-*O*-glucoside (Q3G), kaempferol-3-*O*-glucoside (K3G) and quercetin-3-*O*-rhamnoside (Q3Rha). In contrast, only five of these compounds were detected in F-9, where Q3Rut and CGA were not observed under the analytical conditions used.

Fractions F-5 and F-7 were mainly characterized by the predominance of quercetin derivatives, especially Q3Rha and Q3G. In contrast, F-9 displayed a differentiated profile enriched in M3G and 2,5DHB. These compositional differences may partially explain the distinct antioxidant, enzyme-inhibitory, and cytoprotective responses observed among the fractions.

Overall, the predominance of glycosylated quercetin derivatives and related phenolic compounds is consistent with the strong antioxidant, anti-inflammatory, enzyme-inhibitory, and cytoprotective activities observed for these fractions. Differences in relative composition among F-5, F-7, and F-9 may explain the distinct bioactivity profiles recorded in the in vitro assays.

## 3. Discussion

Free radicals, including ROS and RNS, originate from both endogenous and exogenous sources. Endogenous factors such as inflammation, ischemia, excessive exercise, stress, and aging produce free radicals as metabolic byproducts under physiological conditions. The exogenous sources of ROS include environmental pollutants, heavy metals (e.g., Cd, Pb, and Fe), chemotherapy, organic solvents, heavy smoking, and heavy alcohol consumption, among others [1]. At physiological levels, the ROS play a key role in activating signaling pathways, maintaining redox balance, regulating cellular metabolism, and defending against pathogens. Enzymatic reactions in the respiratory chain, prostaglandin synthesis, phagocytosis, and the cytochrome P450 system primarily produce ROS and RNS. Nonenzymatic reactions, such as the interaction of oxygen with organic compounds or exposure to ionizing radiation, generate free radicals [1,2]. The imbalance between pro-oxidant production and antioxidant defenses results in elevated levels of free radicals and oxidants, which leads to oxidative stress. This process can damage various cellular structures, including membranes, lipids, proteins, and DNA [16,17,18,19,20,21]. Antioxidants reduce oxidative stress by neutralizing free radicals through several mechanisms, including the antioxidant enzymes superoxide dismutase, catalase, and glutathione peroxidase, as well as non-enzymatic antioxidants such as lipoic acid, glutathione, β-arginine, and coenzyme Q10. Additionally, exogenous antioxidants derived from animal and plant sources, obtained through diet or supplementation, can help to reduce oxidative stress [9].

In the present study, we showed that fractions F-1 to F-11 obtained from the bio-guided fractionation of the acetone extract of *B*. × *buttiana* flowers and bracts exhibited concentration-dependent antioxidant activity in the DPPH assay. Although these assays are limited to chemical systems, they provide useful initial insight into the antioxidant capacity of the fractions and support their selection for further biological evaluation. The fractions that exhibited the highest efficacy and potency in DPPH scavenging were F-5, F7, and F-9, as they had the lowest IC_50_ values. The antioxidant capacity of the fractions was strongly correlated with their TPC and TFC values. Therefore, the qualitative and quantitative differences observed among fractions are likely attributable to variations in phenolic acid and flavonoid levels. These findings are consistent with previous studies demonstrating a positive correlation between TPC, TFC, and antioxidant activity in diverse plant extracts [12,15] and further reinforce our previous observations regarding the antioxidant properties of acetone and ethanol extracts of *B*. × *buttiana* (var. rose) [23,26]. To further support these findings, the antioxidant efficiency and yield were analyzed using the AAI, IC_50_/TPC, and RAY-TPC/TPC ratios [31]. The analysis showed that fractions F-5, F-7, and F-9 exhibited very strong antioxidant activity and were therefore selected for HPLC and in vitro assays. The antioxidant activity of these selected fractions was later confirmed in the NO scavenging assay, in which all three displayed comparable NO scavenging inhibition, with F-5 displaying the highest potency (lowest IC_50_). This effect is likely due to the presence of phenolic compounds and flavonoids, which possess strong redox properties [8,9]. These compounds exert their antioxidant effects through multiple mechanisms, including effective hydrogen-atom and electron donation, metal ion chelation, and sequestration of H_2_O_2_. When interacting with reactive species, phenolic compounds form stable radical intermediates via resonance stabilization of their aromatic ring [8,9,15]. Additionally, synergistic interactions with endogenous antioxidants, including ascorbate and tocopherol, enhance overall antioxidant efficacy [9,11,12]. However, the activity of these compounds is context-dependent and varies with concentration and the surrounding chemical environment [13].

HPLC is widely employed for the chemical characterization of extracts from medicinal and food species, revealing chromatographic profiles enriched in glycosylated flavonoids, hydroxybenzoic and hydroxycinnamic acids, and procyanidins, among others [10,14]. In this study, HPLC analysis enabled the identification of glycosylated and aglycone phenolic acids, as well as flavonoids such as quercetin and kaempferol. Fraction F-5 was found to predominantly contain the quercetin glycosides Q3Rha and Q3G, which function as hydrogen donors, although glycosylation can reduce their membrane affinity [13]. Fraction F-7 exhibited a mixture of flavonoids, with higher relative concentrations of Q3Rut, K3G, and M3G [15,16], while F-9 was mainly composed of M3G and 2, 5-DHB, which demonstrated potent metal scavenging and chelating abilities [17,18]. These findings underscore that the antioxidant activity of the fractions varied according to the specific compound profiles. Notably, antioxidant efficacy was influenced not only by the total content of phenolic compounds or flavonoids but also by structural characteristics, polarity, and synergistic interactions, as supported by the AAI and RAY analyses.

ROS are also produced during the inflammatory response and are essential for host defense against pathogens. However, sustained and excessive ROS production leads to oxidative stress, which perpetuates chronic inflammation. This reciprocal interaction between oxidative stress and inflammation contributes directly to the pathogenesis of chronic diseases, such as diabetes, cardiovascular disease, arthritis, and neurodegenerative disorders. ROS act as central mediators, promoting cellular damage and disease progression [3]. Thus, it is likely that the fractions exhibiting strong antioxidant activity also display anti-inflammatory properties. To investigate this relationship, we employed well-established in vitro assays to evaluate the effects of the three selected fractions on the activities of PLA_2_, COX-1, COX-2, and LOX. Our results showed that F-5, F-7, and F-9 significantly inhibited erythrocyte hemolysis and BSA denaturation, confirming their anti-inflammatory potential. Among them, F-5 exhibited the greatest potency, whereas F-7 and F-9 displayed moderate but consistent activity. In addition, all three fractions significantly inhibited key enzymes involved in arachidonic acid metabolism, suggesting that their anti-inflammatory effect may be partially mediated by decreased ROS production resulting from LOX and COX inhibition under inflammatory conditions [34].

Notably, all fractions demonstrated strong selectivity (SI) for COX-2, with SI values of 3.6, 2.7, and 2.9 for F-5, F-7, and F-9, respectively. These results are consistent with and corroborate previous findings using the acetone extract, indicating that the fractions retain the same predominantly anti-inflammatory profile [25]. This selectivity toward COX-2 over COX-1 may reduce the risk of gastric adverse effects compared to COX-1 inhibition. Notably, F-7 was particularly effective in inhibiting COX-2, consistent with the presence of quercetin and kaempferol, which block the enzyme’s hydrophobic channel and prevent arachidonic acid access. F-7 and F-9 displayed the strongest LOX inhibition, which may result from flavonoid chelation of the active-site iron and radical stabilization by myricetin, blocking leukotriene formation [37]. Despite its lower flavonoid content compared to F-7, F-5 exhibited the highest inhibition of BSA, trypsin, and PLA_2_ isoforms (hG-IIA and pG-IB) and LOX. This activity profile suggests a possible specificity of F-5 for proteolytic enzymes and phospholipase-dependent pathways. Taken together, the findings suggest that F-5 has the greatest anti-inflammatory potential among the three fractions.

Furthermore, given that oxidative stress and inflammation are recognized contributors to the onset and progression of type 2 diabetes mellitus and considering the previously reported glucose-lowering effects of this genus, we assessed the potential inhibitory activity of the most active fractions against key enzymes involved in carbohydrate metabolism. Our findings revealed that fractions F-5, F-7, and F-9 also inhibited the key enzymes α-amylase and α-glucosidase, both of which are crucial in blood sugar regulation. Notably, F-7 and F-9 exhibited the lowest IC_50_ values, indicating superior potency in inhibiting these enzymes. This enhanced activity may be attributed to the presence of quercetin and myricetin glycosides, which are known to bind to the catalytic sites of the enzyme, thereby reducing substrate accessibility [35]. These results agree with previous studies reporting that ethanolic extracts of *B. spectabilis* reduced blood glucose levels in diabetic rats [21,22], and provide a basis for future research into the therapeutic potential of *Bougainvillea* species and support their traditional use as antidiabetic agents. Moreover, moderate tyrosinase inhibition was attributed to quercetin glycosides, which chelate copper at the catalytic site and compete with L-DOPA, reducing melanin biosynthesis [38].

The antioxidant relevance of the selected fractions was further validated in a cell-based model of oxidative injury. Hydrogen peroxide markedly compromises cellular viability through intracellular ROS generation, membrane damage, and mitochondrial dysfunction. Pre-treatment of L929 fibroblasts with F-5, F-7, and F-9 fractions significantly preserved cell viability, indicating cytoprotective effects against oxidative stress. Fraction F-5 showed the highest protection, although all three fractions were active. This result is particularly relevant because it reinforces the relevance of the selected fractions beyond cell-free antioxidant systems. The capacity to preserve fibroblast viability may also suggest potential applications in tissue protection and oxidative damage-associated conditions [39].

Altogether, these results indicate that the antioxidant potential of the fractions is enhanced by their ability to inhibit key metabolic and inflammatory enzymes and provide cytoprotection under oxidative stress. These combined effects are likely attributable to the synergistic action of flavonoids and phenolic acids rather than to a single constituent. This dual mechanism, attributed to these compounds, involves radical scavenging and, via hydrogen bonding and hydrophobic interactions, metal-ion chelation. The fractions exhibit a synergistic profile in which antioxidant and enzyme-inhibitory activities converge, thereby strengthening their therapeutic potential against oxidative stress-related and inflammation-related disorders, including chronic diseases such as diabetes mellitus. Consequently, some limitations should be acknowledged: compound assignments were qualitative and tentative based on HPLC-UV-Vis profiling; therefore, confirmatory LC-MS/MS or NMR studies are warranted. Additionally, biological activities were evaluated in vitro, and in vivo pharmacokinetic and efficacy studies are necessary to establish therapeutic relevance.

## 4. Materials and Methods

### 4.1. Chemical Reagents

Acetonitrile, Bovine serum albumin (BSA), Diclofenac sodium salt, 2-[(2,6-Dichlorophenyl)amino] benzeneacetic acid sodium salt, quercetin, quercetin-*O*-glucoside, kaempferol, kaempferol 3-*O*-glucoside, myricetin glucoside, rutin, arachidonic acid, Kojic acid, 2,2-diphenyl-1-picrylhydrazyl (DPPH), sodium hydroxide (NaOH), aluminum chloride (AlCl_3_), sodium nitrite (NaNO_2_), potassium persulfate (K_2_S_2_O_8_), hydrochloric acid (HCl), N-11-naphthyethenediamine, Acarbose, monobasic potassium phosphate, dibasic potassium phosphate, glacial acetic acid, sodium acetate (reagent grades), Tween-20, acetone, and ethanol were acquired from Sigma-Aldrich Chemical Co. (Toluca, EM, Mexico). The COX inhibitor detection assay kit (ovine/human) and PLA_2_ (hG-IIA, pG-IB) from bovine pancreas were obtained from Cayman Chemical Co. (Ann Arbor, MI, USA).

### 4.2. Collection of Vegetal Material

Flowering bracts (289.2 g) were collected in March 2018 in Temixco, Morelos, Mexico (18°52′20.1″ N and 99°14′40.6″ W, at an altitude of 1185 m). A reference specimen was dehydrated and identified as *Bougainvillea* × *buttiana* Holtum & Standl. (var. Rose). The specimen was deposited in the HUMO Herbarium of the Center for Research in Biodiversity and Conservation (CIByC-UAEM), Cuernavaca, Morelos, Mexico, under designation number 33872.

### 4.3. Preparation of the Bougainvillea × Buttiana Acetone Extract and Bioguided Fractionation

Acetone was selected based on previous reports demonstrating its effectiveness for extracting phenolic and flavonoid compounds from plant matrices, particularly compounds with intermediate polarity [23]. Additionally, acetone has been employed in phytochemical and antioxidant studies due to its high extraction efficiency for bioactive secondary metabolites. The extraction procedure and bioguided fractionation were conducted as described in our previous study [23,24]. Briefly, the bracts with flowers were dehydrated at 25 °C and ground into a fine powder. Two grams of this material were subjected to exhaustive maceration in 100% acetone for 72 h. The mixture was filtered using Whatman n° 1 filter paper, and the residue was subjected to three additional extractions. The extracts were combined and concentrated by dehydration using a Büchi^®^ Rotavapor R-100 (Sigma-Aldrich, Toluca, EM, Mexico) at 60 °C. For bioguided fractionation, the extract was subjected to open-column chromatography packed with 60–200 mesh silica gel. Elution was carried out sequentially with solvents of increasing polarity: dichloromethane (DCM) and 100% methanol, followed by washes with water and acetone. The fractions obtained were analyzed by thin-layer chromatography, and those displaying similar chromatographic profiles were pooled. In total, eleven fractions were collected and labeled F-1 to F-11.

### 4.4. Quantification of Total Phenolic Content and Total Flavonoid Content

#### 4.4.1. Total Phenolic Content (TPC)

The total phenolic content (TPC) of each fraction was quantified by the Folin–Ciocalteu assay [40]. In brief, 100 μL of each fraction (equivalent to 100 µg) was mixed with 0.5 mL of Folin–Ciocalteu reagent and 1 mL of 7.5% (*w*/*v*) sodium carbonate, then brought to a final volume of 2 mL. The mixture was stirred and allowed to react for 30 min before absorbance was measured at 765 nm. TPC was expressed as milligrams of gallic acid equivalents per gram of fraction (mg GAE/g fraction). Quantification was based on the calibration curve equation: Y = 8.768X − 0.027 (R^2^ = 0.994), where X is the absorbance and Y is mg GAE/g of fraction. All analyses were performed in triplicate.

#### 4.4.2. Total Flavonoid Content (TFC)

The total flavonoid content (TFC) of each fraction was measured following the method published by Zhishen et al. (1999) [41]. Briefly, 500 µL of each fraction was combined with 75 µL of 5% NaNO_2_ solution and maintained at 25 °C for 6 min. Subsequently, 150 µL of 10% AlCl_3_·6H_2_O solution was added, and the mixture was incubated for an additional 5 min. Next, 500 µL of 1 M NaOH and 2500 µL of distilled water were added, and the mixture was thoroughly mixed. The absorbance was then measured at 510 nm using a spectrophotometer. All measurements were conducted in triplicate. The TFC was expressed as milligrams of quercetin equivalents per gram of fraction (mg QE/g fraction) using the calibration curve equation: Y = 2.398X + 0.884 (R^2^ = 0.995), where X is absorbance and Y is mg QE/g fraction.

### 4.5. Antioxidant Activity

Antioxidant potential of each selected fraction was determined in vitro using the 1,1-diphenyl-2-picrylhydrazyl (DPPH) and Nitric Oxide (NO) radical-scavenging assays.

#### 4.5.1. DPPH Free Radical Scavenging

The antioxidant capacity of each selected fraction was evaluated using the standard DPPH (2,2-diphenyl-1-picrylhydrazyl) assay [30]. This method quantifies radical scavenging activity by measuring the reduction of the stable DPPH• radical (violet) to diphenylpicrylhydrazyl (yellow), monitored as a decrease in absorbance at 517 nm. Fractions at concentrations of 0, 2.5, 25, 50, 100, 200, 300, 400, and 500 µg/mL were tested. The sample preparation, consisting of equal volumes of each fraction at the specified concentration and 0.1 mM methanolic DPPH solution, was mixed and then incubated in the dark at 25 °C for 30 min. Absorbance at 517 nm was measured for both the samples and the control (methanolic DPPH solution) using a UV-visible spectrophotometer. Quercetin was used as the standard, and all analyses were carried out in triplicate. For the construction of the quercetin calibration curve, the same concentration range as the sample of selected fractions was used, which was defined by the equation Y = −6.564X + 1.578 (R^2^ = 0.994). The antioxidant activity was expressed as the percentage of DPPH radical scavenging, calculated as follows:

$$DPPH\;scavenging\;activity\;\%=\frac{\left({Abs}_{Control}-{Abs}_{Sample}\right)}{{Abs}_{Control}}\times 100$$

#### 4.5.2. NO Nitric Oxide Radical Scavenging Assay

The nitric oxide (NO) radical-scavenging assay was performed using the Griess reaction, as described in the standard procedure [32,33]. The reaction mixture containing 2 mL of 10 mM sodium nitroprusside prepared in 10 mM phosphate-buffered saline (PBS; pH 7.3) and 0.5 mL of the standard solution reference (gallic acid) or the fractions (0.01, 0.1, 1, and 2 mg/mL) was incubated with continual shaking (70 rpm) at 25 °C for 180 min. For this assay, the reference standard (Gallic acid, 0–150 µg/mL) was used to generate the calibration curve. After incubation, 0.1 mL aliquots were transferred to 96-well plates and mixed with 0.1 mL of Griess reagent (1% sulfanilamide in 3% phosphoric acid and 1% N-(1-naphthyl)ethylenediamine). The plates were maintained at room temperature for 5 min. Absorbance was measured at 540 nm, and all assays were performed in triplicate. Controls contained no test fraction and represented 100% nitrite formation. Antioxidant activity (AA) was calculated as follows:

$$Inhibition\;NO\;scavenger\;activity\;\%=\frac{\left(A_{0}-A_{1}\right)}{A_{0}}\times 100$$

where A_0_ is the control absorbance, and A_1_ is the fraction absorbance.

#### 4.5.3. Selection of Fractions with the Best Antioxidant Activity

To identify the fractions with the best antioxidant activity to be evaluated in further analysis, the antioxidant activity index (AAI) and the IC_50_/TPC, IC_50_/TFC, and the Relative Antioxidant Yield (RAY)-TPC or -TFC ratios were calculated. (AAI) was estimated by dividing the final concentration of DPPH (µg/mL) by the IC_50_ (µg/mL) of each fraction. Values are interpreted as follows: poor activity (<0.5), moderate (<1.0), strong (<2.0), and very strong (>2.0), as proposed by Scherer and Godoy (2009) [31]. The IC_50_/TPC and IC_50_/TFC ratios represent the amounts of phenolic or flavonoid compounds required to achieve 50% antioxidant activity and were calculated by simple division. IC_50_/TPC ratios below 10 reflect high efficiency, values from 10 to 30 indicate moderate efficiency, and values above 30 indicate low efficiency. RAY-TPC and RAY-TFC ratios were calculated based on the relationship between the antioxidant activity index (AAI) values × 100 and total phenolic or flavonoid content (RAY-TPC) or (RAY-TFC). Lower RAY values correspond to higher antioxidant performance. A RAY-TPC ratio greater than 2.0 indicates high efficiency, 0.5–2.0 indicates moderate efficiency, and values below 0.5 represent low efficiency.

### 4.6. In Vitro Anti-Inflammatory Activity Assays

The anti-inflammatory potential of fractions F-5, F-7, and F-9 was evaluated in vitro using biochemical assays: erythrocyte membrane stabilization, heat-induced denaturation of bovine serum albumin (BSA), and trypsin inhibition. To clarify the mechanism, inhibition of key inflammatory enzymes, including cyclooxygenases (COX-1 and COX-2), lipoxygenases (LOX), and phospholipase A_2_ (sPLA_2_), was also assessed. All assays were conducted in triplicate at concentrations of 100, 200, 300, 400, and 500 µg/mL.

#### 4.6.1. Erythrocyte Membrane Stabilization Assay

The membrane-stabilizing activity of selected fractions F-5, F-7, and F-9 was calculated using a two-step erythrocyte membrane stabilization assay, as previously described [42]. Briefly, 5 mL of mouse blood was collected by venipuncture into EDTA-containing tubes. The samples were centrifuged at 3000 rpm for 10 min at 25 °C, and the plasma was discarded. Packed erythrocytes were washed three times with an equal volume of isosaline solution (0.85% *w*/*v* NaCl) until the supernatant was clear. A 10% erythrocyte suspension was prepared in isosaline. For the assay, aliquots of the erythrocyte suspension were incubated with the test fractions or diclofenac (positive control) in hyposaline solution at 56 °C for 30 min. Following incubation, hemolysis was assessed by measuring the absorbance of the supernatant at 540 nm. The membrane stabilization % was calculated as follows:

$$Membrane\;stabilization\;\%=1-\left(\frac{{Abs}_{fraction}}{{Abs}_{control}}\right)\times 100$$

#### 4.6.2. BSA Denaturation Assay

The inhibitory effects of fractions F-5, F-7, and F-9 on heat-induced BSA denaturation were evaluated using the method reported by Chandra et al. (2012) and adapted to our laboratory conditions [43]. The reaction mixtures contained varying amounts of each selected fraction or of the reference drug, diclofenac, in phosphate-buffered saline (PBS, pH 6.4), while control samples contained PBS only. The mixtures were incubated at 37 °C for 20 min, then at 70 °C for 5 min to denature the proteins. After cooling to room temperature, absorbance was evaluated at 660 nm using a UV-visible spectrophotometer. The control sample (without any test compound) was used as a 100% protein-denaturation reference. The percentage inhibition of BSA denaturation was calculated using the formula:

$$\%\;inhibition\;of\;BSA\;denaturation=100-\left[\left(1-\frac{{Absorbance}_{test\;fraction}}{{Absorbance}_{control}}\right)\times 100\right]$$

#### 4.6.3. Trypsin Inhibition Activity Assay

Proteinase inhibitory activity of each fraction, F-5, F-7, and F-9, was evaluated using the trypsin inhibition method [25]. Various concentrations of each fraction (100, 200, 300, 400, and 500 μg/mL) or diclofenac as the reference drug. For the assay, 1 mL of trypsin solution (0.06 mg in 20 mM Tris-HCl buffer) was incubated for 15 min. at 37 °C. Afterwards, 1 mL of 0.8% (*w*/*v*) casein solution was added, and the mixtures were incubated for an additional 20 min. at 37 °C. Finally, the reaction was interrupted by adding 2 mL of 70% perchloric acid, followed by centrifugation at 3000 rpm for 5 min. The absorbance of the resulting supernatants was measured at 210 nm. A reaction mixture without fractions was prepared as the control (blank). The percentage inhibition was estimated using the following formula:

$$\%inhibition\;of\;denaturation=\left(1-\frac{{Absorbance}_{test\;fraction}}{{Absorbance}_{control}}\right)\times 100$$

#### 4.6.4. Inhibition of the Activity of Inflammation-Related Enzymes

PLA_2_: The inhibitory effects of selected fractions on sPLA_2_ activity were assessed using a previously described protocol [25], employing the hG-IIA and pG-IB isoforms of group IIA sPLA_2_. The substrate buffer was prepared by dissolving lecithin (3.5 mM), sodium taurodeoxycholate (3 mM), and CaCl_2_ (10 mM) in NaCl (100 mM), adding phenol red (0.055 mM), and adjusting the pH to 7.6. For each assay, 10 µL of each fraction at concentrations of 100, 200, 300, 400, and 500 µg/mL were mixed with 10 µL of PLA_2_-GIB, then incubated at 25 °C for 20 min. Subsequently, 1 mL of the PLA_2_ substrate was added, and hydrolysis kinetics were monitored over 5 min by measuring absorbance at 558 nm. The percentage inhibition was determined by comparing the residual activity of each sample to that of the negative control (without fraction).

COX-1 and COX-2: The inhibitory effects of the fractions on COX-1 and COX-2 enzymatic activity were assessed using a commercial ovine/human COX assay kit, following the manufacturer’s protocol [25]. The assay quantifies PGF2α production, generated by reducing COX-derived PGH2 with SnCl_2_. For COX-2 measurements, 105 µL of reaction buffer, 10 µL of COX-1/2 enzymes, and 20 µL of each test fraction were combined. Enzymes were inactivated by boiling the samples for 3 min. The reaction was started by adding 10 µL of arachidonic acid, 50 µL of 1 M HCl, and 100 µL of SnCl_2_. Absorbance was measured at 405 nm for each mixture containing a test fraction or diclofenac, the reference drug. Inhibitory activity was reported as a percentage inhibition compared to the control.

LOX: The anti-LOX activity of the fractions was evaluated using a previously published spectrophotometric method [44]. Lipoxygenase (LOX) catalyzes the oxidation of polyunsaturated fatty acids, such as linoleic acid, producing conjugated diene hydroperoxides detectable at 234 nm. The control positive drug was nordihydroguaiaretic acid (NDGA). LOX inhibition was measured by recording the absorbance at 234 nm immediately after adding the test fraction and comparing the change to that of the control. The percentage of inhibition was calculated as follows:

$$\%\;inhibition\;of\;LOX\;activity=\left(\frac{{Absorbance}_{Control}-{Absorbance}_{sample}}{{Absorbance}_{Control}}\right)\times 100$$

### 4.7. Inhibition of the Activity of Enzymes Related to Diabetes

To determine if the acetone extract of *B.* × *buttiana* has antidiabetic potential, the effect of selected fractions on the activity of the enzymes α-amylase, α-glucosidase, and tyrosinase was evaluated in vitro.

α-Amylase: The α-amylase inhibitory activity of the fractions was assessed using a colorimetric assay with soluble starch and dinitrosalicylic acid (DNS), as described by Liu et al. 2014 [45]. In this procedure, 40 μL of α-amylase solution (5 U/mL), 0.36 mL sodium phosphate buffer (0.02 M, pH 6.9, 6 mM NaCl), and 0.2 mL of each test fraction or acarbose (0.5, 1.0, 1.5, or 2.0 mg/mL) were incorporated and incubated for 20 min at 37 °C. The reaction was initiated by adding 300 μL of 1% starch solution in the same buffer, followed by a further 20 min incubation at 37 °C. The reaction was interrupted by adding 0.2 mL of DNS reagent, and the mixture was heated to boiling for 5 min. After cooling to room temperature, the mixture was diluted with 10 mL of distilled water, and absorbance was measured at 540 nm using a UV-visible spectrophotometer. The percentage of enzyme inhibition was calculated as:

$$\mathrm{Inhibitory}\;\mathrm{activity}\;\%=\left(\frac{{Absorbance}_{Control}-{Absorbance}_{sample}}{{Absorbance}_{Control}}\right)\times 100$$

α-Glucosidase: The α-glucosidase inhibitory activity of the fractions was evaluated using a colorimetric assay with p-nitrophenyl-α-D-glucopyranoside (pNPG), as previously described [46]. Briefly, 10 μL of α-glucosidase solution (1 U/mL) was added to a 96-well plate with 60 μL of phosphate buffer (0.1 mM, pH 6.8) and 100 μL of each test fraction, acarbose (0.5, 1.0, 1.5, or 2.0 mg/mL), or a negative control. After a 10 min pre-incubation at 37 °C, 30 μL of 2 mM pNPG was added to start the reaction. Absorbance at 405 nm was measured every 15 min for 2 h to monitor p-nitrophenol release. Enzyme inhibition was determined by calculating the area under the curve (AUC) for each condition, and the inhibitory effect (%) was calculated as:

$$\mathrm{Inhibition}\;\mathrm{of}\;\alpha \text{-}\mathrm{Glucosidase}\;\%=\left(\frac{{Absorbance}_{Control}-{Absorbance}_{sample}}{{Absorbance}_{Control}}\right)\times 100$$

Tyrosinase: The tyrosinase-inhibitory activity of the fractions was evaluated using an L-DOPA oxidation assay in 96-well plates, following the method described by Zuo et al. (2018) [47]. Briefly, 40 μL of tyrosinase (100 U/mL) was mixed with 80 μL of phosphate buffer (0.1 M, pH 6.8) and 80 μL of each fraction or kojic acid (positive control). The plate was incubated at 25 °C for 10 min. The reaction was initiated by adding 40 μL of 2 mM L-DOPA. Absorbance was determined at 475 nm every 5 min for 30 min using a microplate reader. The inhibition percentage was calculated as:

$$\mathrm{Inhibition}\;\mathrm{of}\;\mathrm{Tyrosinase}\%\;=\left(\frac{{Absorbance}_{Control}-{Absorbance}_{sample}}{{Absorbance}_{Control}}\right)\times 100$$

### 4.8. Hydrogen Peroxide (H_2_O_2_)-Induced Oxidative Stress in L929 Cells and Evaluation of Survival

To evaluate the cytoprotective effect of the extracts, hydrogen peroxide was used to induce oxidative stress, as previously described by Balekar et al. (2012) [48]. Mouse fibroblast cells (ATCC clone L929) were cultured in Dulbecco’s Minimum Essential Medium (DMEM) supplemented with 10% Fetal Bovine Serum (FBS), 100 U/mL penicillin, 100 mg/L streptomycin, and 500 mg/L neomycin. Briefly, the L929 cells were seeded at a density of 5 × 10^3^ cells/well in DMEM supplemented with 10% FBS and incubated at 37 °C with 5% CO_2_ for 18 h. To determine the hydrogen peroxide (H_2_O_2_) concentration capable of inducing oxidative damage, cells were exposed to 0–1.0 mM, and cell viability was assessed after 24 h using the MTT assay. The concentration of H_2_O_2_ selected for the assays was 1.0 mM, since it decreased viability by 50–80%. For the cytoprotective assays, after the initial incubation period, the medium was replaced with fresh medium containing 0–200 µg/mL of the F-5, F-7, and F-9 fractions under pre-, co-, and post-treatment conditions. Subsequently, part of the medium was removed, 50% DMSO was added to dissolve formazan crystals, and absorbance at 540 nm was measured using a microplate reader. The results were expressed as percentage viability relative to control (100% viability).

### 4.9. Analysis by High-Performance Liquid Chromatography (HPLC-UV-Vis)

The chromatographic analysis of fractions F-5, F-7, and F-9 was executed in a Waters 2695 separation module system with a Waters 2695 photodiode matrix detector and Pro Empower^TM^ 3software (Waters Corporation, Milford, MA, USA). The chemical separation was performed by using a Supelcosil LC-F column (4.6 mm × 250 mm i.d., particle size 5 μm) (Sigma-Aldrich, Bellefonte, PA, USA). The mobile acid phase was performed using 0.5% trifluoracetic aqueous solution (solvent A) and acetonitrile (solvent B) gradient: 0–1 min, 0% of B; 2–3 min, 5% of B; 4–20 min, 30% of B; 21–23 min, 50% of B; 24–25 min, 80% of B; 26–27 min, 100% of B; 28–30 min, 0% of B. The flow rate was 0.9 mL/min with a volume of 10 μL sample, and the absorbance was evaluated at 270 nm. A preliminary identification of the resolved peaks was performed by comparison with the retention times (tR) and UV-Vis characteristic bands of known standards and the literature. The results are expressed as % of the relative area. Unidentified peaks contributed to the qualitative chromatographic profile but were excluded from quantitative analysis. All analyses were conducted at the Southern Biomedical Research Center (CIBIS-IMSS).

## 5. Conclusions

This study highlights the complementary bioactivities of the analyzed fractions. Among the eleven fractions evaluated, F-5, F-7, and F-9 exhibited the highest antioxidant performance, which was strongly associated with their elevated phenolic and flavonoid contents. These fractions effectively scavenged DPPH and nitric oxide radicals, confirming their redox-modulating capacity. Fraction F-5, which is rich in quercetin glycosides, exhibits strong antioxidant potential via radical scavenging, hydrogen donation, and metal chelation. In contrast, fractions F-7 and F-9, which contain kaempferol and myricetin derivatives, display pronounced anti-inflammatory activity through selective COX-2 and LOX inhibition, enzyme modulation, and radical stabilization. Notably, all three fractions protected erythrocytes against hemolysis, demonstrating their ability to preserve cell membrane integrity under oxidative stress. Fractions F-5, F-7, and F-9 also significantly protected L929 fibroblasts against H_2_O_2_-induced oxidative damage, supporting their cytoprotective potential in a cellular model. Qualitative tentative HPLC-UV-Vis characterization indicated that these bioactivities are likely associated with the presence of glycosylated flavonoids and phenolic acids, particularly quercetin derivatives, myricetin glycosides, kaempferol derivatives, chlorogenic acid, and related compounds. Collectively, these findings indicate that F-5 may function as a natural antioxidant, whereas F-7 and F-9 show promise as anti-inflammatory agents, either individually or in combination. Further in vitro and in vivo investigations are needed to confirm these biological effects, elucidate bioavailability and mechanisms of action, and support the future translational applications of these fractions for the prevention or management of oxidative stress- and anti-inflammation-related disorders, particularly in the context of complementary and/or orthomolecular medicine.

## Figures and Tables

**Table 1:** Inhibitory effects of fractions F-5, F-7, and F-9 on enzymes related to inflammation and carbohydrate metabolism.

Class of Enzyme	Enzyme	Reference Drug	Fraction IC_50_ (µg/mL)		
		IC_50_ (µg/mL)	F-5	F-7	F-9
**Inflammation**- **related enzymes**	PLA_2_ hG-IIA	**Indomethacin** 91.11 ± 4.67	160.00 ± 8.00 ***	230.00 ± 11.00 ***	235.00 ± 11.98 ***
	PLA_2_ pG-IB		190.00 ± 9.00 ***	280.0 ± 14.00 ***	310.0 ± 14.60 ***
	COX-1	**Diclofenac** 140.12 ± 7.38	1258.33 ± 61.77 ***	749.51 ± 37.49 ***	962.11 ± 48.11 ***
	COX-2	**Diclofenac** 78.00 ± 3.86	347.17 ± 17.33 ***	292.64 ± 14.66 ***	327.58 ± 16.32 ***
	LOX	**NDGA** 56.10 ± 2.88	111.00 ± 4.32 ***	98.30 ± 3.93 ***	120.70 ± 5.19 ***
**Diabetes**- **related** **enzymes**	α-Amylase	**Acarbose** 14.81 ± 0.83	13.18 ± 0.52 **^ns^**	11.54 ± 0.48 **	11.94 ± 0.51 **
	α-Glucosidase		16.80 ± 0.73 ***	12.30 ± 0.51 ***	12.22 ± 0.42 **
	Tyrosinase	**Kojic acid** 12.50 ± 0.53	85.00 ± 3.65 ***	76.50 ± 3.31 ***	83.55 ± 3.76 ***

Notes: Values are expressed as mean ± SD (*n* = 3). Lower IC_50_ values indicate stronger inhibitory activity. Values in bold indicate the most active fraction for each target enzyme. Diclofenac was used as a reference inhibitor for inflammation-related assays; acarbose for α-amylase and α-glucosidase; kojic acid standard for tyrosinase, and NDGA for LOX. ** *p* < 0.01, *** *p* < 0.001 compared to the IC_50_ of the reference drug by one-way ANOVA followed by the Dunnett test. ns: not significant.

**Table 2:** Area Percentage of compounds preliminarily identified by HPLC in fractions F-5, F-7, and F-9.

Compound	Claves	% Area		
		F-5	F-7	F-9
2,5dihydroxybenzoic acid	2,5DHB	5.58	1.07	17.73
Myricetin 3-*O*-beta-D-glucopyranoside	M3G	2.14	6.03	25.84
Chlorogenic acid	CGA	3.96	2.71	ND
Quercetin-3-rutinoside	Q3Rut	5.35	8.59	ND
Quercetin-3-*O*-glucoside	Q3G	30.43	30.09	23.71
Kaempferol-3-*O*-glucoside	K3G	2.34	3.37	2.61
Quercetin-3-*O*-rhamnoside	Q3Rha	42.42	35.78	14.17

Notes: Values correspond to relative peak area percentages of identified peaks within each chromatogram. ND: not detected under the chromatographic conditions used. Compound assignments are tentative and based on retention time, UV-Vis spectra, and comparison with available standards and literature data.

## Data Availability

The data are contained in the article.

## Abbreviations

The following abbreviations are used in this manuscript:

2,5DHB	2,5-dihydroxybenzoic acid
AAI	Antioxidant Activity Index
AlCl_3_	Aluminum Chloride
BSA	Bovine Serum Albumin
CGA	Chlorogenic Acid
COX	Cyclooxygenase
DCM	Dichloromethane
DPPH	2,2′-diphenyl-1-picrylhydrazine
F-1 to F11	Fraction 1, Fraction 2, Fraction 3, etc.
GAE	Gallic Acid Equivalents
HCl	Hydrochloric Acid
HPLC	High-performance liquid chromatography
IC_50_	Half-maximal inhibitory concentration
K_2_S_2_O_8_	Potassium Persulfate
K3G	Kaempferol-3-*O*-glucoside
LOX	Lipoxygenase
MeOH	Methanol
M3G	Myricetin 3-*O*-beta-D-glucopyranoside
NaOH	Sodium Hydroxide
NaNO_2_	sodium nitrite
PBS	Phosphate-buffered saline
PLA_2_	Phospholipase A_2_
pNP	p-nitrophenol
pNPG	p-nitrophenyl-α-D-glucopyranoside
Q3G	Quercetin-3-*O*-glucoside
Q3Rha	Quercetin-3-*O*-rhamnoside
Q3Rut	Quercetin-3-rutinoside
RAY	Relative Antioxidant Yield
SD	Standard deviation
SI	Selective index
TFC	Total Flavonoid Content
TPC	Total Phenolic Content
